# The *Orientia tsutsugamushi* ScaB Autotransporter Protein Is Required for Adhesion and Invasion of Mammalian Cells

**DOI:** 10.3389/fmicb.2021.626298

**Published:** 2021-02-04

**Authors:** Yen Thi Hai Nguyen, Chaewon Kim, Yuri Kim, Kyeongseok Jeon, Hong-il Kim, Na-Young Ha, Nam-Hyuk Cho

**Affiliations:** ^1^Department of Microbiology and Immunology, Seoul National University College of Medicine, Seoul, South Korea; ^2^Department of Biomedical Sciences, Seoul National University College of Medicine, Seoul, South Korea; ^3^Institute of Endemic Diseases, Seoul National University Medical Research Center and Bundang Hospital, Seoul, South Korea

**Keywords:** scrub typhus, autotransporter, adhesion, invasion, vaccine

## Abstract

Autotransporter proteins are widely present in Gram-negative bacteria. They play a pivotal role in processes related to bacterial pathogenesis, including adhesion, invasion, colonization, biofilm formation, and cellular toxicity. Bioinformatics analysis revealed that *Orientia tsutsugamushi*, the causative agent of scrub typhus, encodes six different autotransporter genes (*scaA*–*scaF*). Although four of these genes (*scaA*, *scaC*, *scaD*, and *scaE*) are present in diverse strains, *scaB* and *scaF* have been detected in only a limited number of strains. Previous studies have demonstrated that ScaA and ScaC are involved in the adherence of host cells. However, the putative function of other *O. tsutsugamushi* Sca proteins has not been studied yet. In this study, we show that *scaB* is transcribed and expressed on the surface of *O. tsutsugamushi* Boryong strain. Using a heterologous *Escherichia coli* expression system, we demonstrated that ScaB-expressing *E. coli* can successfully mediate adherence to and invasion into non-phagocytic cells, including epithelial and endothelial cells. In addition, pretreatment with a recombinant ScaB polypeptide inhibits the entry of *O. tsutsugamushi* into cultured mammalian cells. Finally, we also identified the *scaB* gene in the Kuroki and TA686 strains and observed high levels of sequence variation in the passenger domains. Here, we propose that the ScaB protein of *O. tsutsugamushi* can mediate both adhesion to and invasion into host cells in the absence of other *O. tsutsugamushi* genes and may play important roles in bacterial pathogenesis.

## Introduction

Scrub typhus is a re-emerging vector-borne disease prevalent in the Asia-Pacific region (Kelly et al., 2009; Kala et al., 2020; Richards and Jiang, 2020). It is caused by an obligate intracellular Gram-negative bacterium, namely *Orientia tsutsugamushi*, which is transmitted to mammalian hosts, including rodents and humans, *via Leptotrombidium* mites at their larval stage (Seong et al., 2001). The clinical symptoms of scrub typhus are highly variable, ranging from mild to fatal infections. Approximately 1–2 weeks after being bitten by the infected vector, patients present with fever, headache, rash, nausea, lymphadenopathy, and eschar at the bite site (Ponnusamy et al., 2018; Kala et al., 2020). Delayed treatment with appropriate antibiotics, such as tetracycline, chloramphenicol, and macrolides, often results in pneumonitis, meningitis, myocarditis, acute renal failure, and acute respiratory distress syndrome. The mortality rate of scrub typhus in the pre-antibiotic era reached up to 40–50% (Taylor et al., 2015).

More than a million cases are estimated to occur annually and a billion individuals are under risk of suffering this disease (Kelly et al., 2009, 2015). In addition, accumulating reports have shown confirmed or suspected scrub typhus cases occurring outside the traditional endemic regions, such as in South America, the Arabian Peninsula, and Africa (Weitzel et al., 2016). Moreover, rodents infected with bacterial strains closely related to *O. tsutsugamushi* have also been found in Africa and Southern Europe (Izzard et al., 2010; Cosson et al., 2015; Kolo et al., 2016; Masakhwe et al., 2018). These reports suggest that the disease is no longer restricted to its endemic territory and is widely distributed. These findings also indicate the existence of unidentified antigenically different strains may exist (Ghorbani et al., 1997; Izzard et al., 2010; Cosson et al., 2015; Weitzel et al., 2016; Jiang and Richards, 2018).

Despite the growing risk and wide spread of the disease, there is no vaccine available to prevent it. Although various types of vaccine candidates have been extensively studied, most of them induce only short-term immunity to their homologous strain (Chattopadhyay and Richards, 2007; Valbuena and Walker, 2012). Therefore, further identification of potential targets as vaccine antigens is required to develop effective vaccines.

*Orientia tsutsugamushi* is an obligate intracellular bacterium that must be internalized into host cells for propagation of infection. Although the precise mechanism of host cell invasion is poorly characterized, several bacterial proteins have been shown to be necessary for attachment and entry into host cells. Previously, we reported that one of the major bacterial outer membrane proteins, a 56 kDa type-specific antigen (TSA56), mediates cellular adherence to or invasion into non-phagocytic cells by interacting with fibronectin (Lee et al., 2008; Cho et al., 2010b). Additionally, we found that there were five different autotransporter proteins (ScaA–ScaE) encoded in the genomes of various strains and defined as Va subclass based on protein 3D structure prediction (Ha et al., 2013). Recently, another group identified a sixth autotransporter gene, *scaF*, in some strains; however, its structure has not yet been classified (Ha et al., 2012; Koralur et al., 2018).

Bacterial autotransporter proteins have been demonstrated to play a role during infection. In general, autotransporter proteins are widely found in Gram-negative bacteria and possess unique structural properties, with their structure consisting of a signal peptide, an N-terminal passenger domain, and a C-terminal autotransporter domain. The passenger domain is translocated through the autotransporter domain and exposed to the extracellular environment, conferring multiple virulence functions, including adhesion, invasion, colonization, biofilm formation, and cellular toxicity (Henderson and Nataro, 2001; Nishimura et al., 2010; Meuskens et al., 2019). Autotransporter genes, including *scaA*, *scaC*, *scaD*, and *scaE* genes, have been detected in most strains of *O. tsutsugamushi*, whereas *scaB* and *scaF* have only been found in a few strains. Previously, we showed that *scaA* and *scaC* are transcribed and actively expressed on the bacterial surface (Ha et al., 2011, 2015). ScaA functions as an adhesion molecule and induces an antibody response that can neutralize the bacterial entry into the host cell, whereas ScaC mediates only adherence, but not invasion into the host cells. Preincubation of host cells with recombinant ScaC significantly inhibited their interaction with *O. tsutsugamushi* (Ha et al., 2011, 2015). Considering the functional role of these autotransporters in *O. tsutsugamushi* and their genetic distribution among various strains, further investigation of the biological effects of other Sca proteins needs to be performed.

In this study, we investigated the function of the ScaB protein of *O. tsutsugamushi*. We found that ScaB expression in *Escherichia coli* was sufficient to mediate both bacterial adherence to and invasion into non-phagocytic host cells, and preincubation of mammalian cell cultures with the ScaB protein inhibited bacterial invasion. In addition, we newly detected the presence of the *scaB* gene in the Kuroki and TA686 strains and observed high levels of sequence variation in the passenger domains. Taken together, these results demonstrate that *O. tsutsugamushi* utilizes ScaB as a ligand protein for the adhesion and invasion of host cells and suggest that ScaB may play an important role in bacterial pathogenesis.

## Materials and Methods

### Ethics Statement

Ethical approval for this work was granted by the Institutional Review Boards of Seoul National University Hospital (IRB 1308-058-513). Animal experiments were approved by the Seoul National University Hospital Institutional Animal Care and Use Committee (SNU-180727-6-7) and performed in strict accordance with the recommendations in the National Guide Line for the care and use of laboratory animals.

### Cell Lines

L929 cells (ATCC NCTC929, American Type Culture Collection), HeLa cells (ATCC CCL-2), A549 cells (ATCC CCL-185), and ECV304 (ATCC CRL-1998), an endothelial cell-like cell line, were maintained in DMEM (Welgene, Daegu, Korea) supplemented with 10% heat-inactivated fetal bovine serum (FBS; Welgene), 100 U/ml penicillin, and 100 μg/ml streptomycin (Gibco BRL) at 37°C in 5% CO_2_.

### Preparation of *O. tsutsugamushi*

*Orientia tsutsugamushi* strains Boryong (AM494475) and Kuroki (M63380) were semi-purified as previously described (Ha et al., 2011). Briefly, when more than 90% of the cells were infected, as determined by an indirect immunofluorescence antibody technique, the cells were collected, homogenized using a glass Dounce homogenizer (Wheaton, Inc., Millville, NJ, United States), and centrifuged at 500 × *g* for 5 min (Kim et al., 2019). The supernatant was stored in liquid nitrogen until use.

### Reverse Transcription-PCR

To detect *O. tsutsugamushi scaB* gene expression, total RNA was isolated from *O. tsutsugamushi*-infected L929 cells by using an RNeasy minikit (Qiagen, Hilden, Germany). The RNA samples were then digested with RNase-free DNase (Qiagen, Hilden, Germany) at room temperature to remove any contaminating DNA. Reverse transcriptase PCR (RT-PCR) was performed using a reverse transcription system (iNtRON Biotechnology, Seongnam, South Korea) and an AccuPower® Taq PCR PreMix kit (Bioneer, Daejon, South Korea). The reactions were performed according to the manufacturers’ instructions by using 5 μg total RNA and 5 μM concentrations of the relevant primers listed in Table 1.

**Table 1:** Primers used in this study.

Primer direction	Primer sequence	Product size (bp) [amplified region (nt position)]	In this study
Forward	GGCGGATCCATGTTAAAAACCAACAAA	1,950 (1–1,950)	RT-PCR, sequencing
Reverse	CGGTCGACCTAGAAATTAGCTTTTAT		
Forward	GGCGGATCCAGTACAACTCAAAGGATATTAGG	1,041 (70–1,116)	Recombinant protein production
Reverse	CGGTCGACACTACTACAAATGTTTGATCC		
Forward	AAAGGATCCACTAAACAAAGCAAGTTT	1,830 (118–1,947)	Adhesion/invasion assay
Reverse	AAACTCGAGGAAATTAGCTTTTATATT		

Restriction enzyme sites are underlined.

### Cloning and Expression of ScaB

The full-length of *scaB* gene (nucleotide 1–1,950) was amplified from the genomic DNA of the *O. tsutsugamushi* Boryong and Kuroki genotype by using the primer pairs listed in Table 1. The PCR product from Kuroki genotype is directly cloned into pCR™2.1 vector for sequencing. To generate the recombinant ScaB protein, a gene fragment corresponding to the ScaB passenger domain (amino acids 23–372) was amplified from the Boryong genomic DNA by using the primer pair listed in Table 1. The amplified fragment was then directionally cloned into pET28a or pGEX4T-1 vector (Novagen, Gibbstown, NJ, United States).

For the expression and purification of ScaB proteins, *E. coli* BL21 (DE3; Novagen) was transformed with ScaB passenger domain then following induction with isopropyl β-D-thiogalactoside (IPTG; 0.1 mM, Duchefa, Zwijndrecht, Netherlands) at 16°C for 16 h, the proteins were purified using Ni-nitrilotriacetic acid His-resin (Qiagen, Carlsbad, CA, United States) or glutathione-Sepharose 4Bcolumns (GE Healthcare, Piscataway, NJ, United States) according to manufacturer’s instructions. The purified proteins were dialyzed against phosphate-buffered saline (PBS) in a Slide-A-Lyzer Dialysis Cassette (Thermo scientific, Rockford, IL, United States) at 4°C for overnight.

### Sequence Analysis

Nucleotide sequences of *scaB* genes amplified from Kuroki genotype were deposited to GenBank under accession no. MW216678. Sequences of *scaB* genes from various genotypes (AM494475.1 for Boryong, LAOM01000054.1 for Sido, LAOA01000033.1 for TA716 genotype, and SPR13699.1 for TA686) were also used for comparative analysis. Nucleotide sequence alignments for constructing phylogenetic trees were processed by Clustal W with maximum likelihood method implemented in MEGA6 software (Tamura et al., 2013). The similarity and identity of those nucleotides and amino acids were calculated through Matrix Global Alignment Tool (MatGAT) version 2.03 (Campanella et al., 2003). The aligned nucleotide sequences were evaluated in SimPlot version 3.5.1 with Kimura (2-parameter) and Empiric Ts/Tv ratio settings (Lole et al., 1999). The aligned amino acid sequences were analyzed through the BLOSUM62-referenced 100 amino acid sliding window analysis. The output values were calculated from R-Project^1^ (Ha et al., 2015; Kim et al., 2017). Line graphs were visualized by GraphPad Prism software version (Graph-Pad Software Inc., La Jolla, CA, United States). For protein structural prediction, HHPred,^2^ PSIPRED,^3^ and I-TASSER^4^ were used (Söding et al., 2005; Buchan et al., 2013; Yang and Zhang, 2015; Buchan and Jones 2019).

### Antibodies and Reagents

Both preimmune and anti-ScaB polyclonal mouse serum (produced by immunization with purified ScaB_24–372_ protein; Koma Biotech, Seoul, South Korea) were used for the experiments. Six week old female C57BL/6 mice (Orientt Bio Inc., Seongnam, South Korea; *n*=5) were immunized subcutaneously three times with 2 weeks interval. Ten microgram of purified ScaB protein in PBS emulsified 1:1 with 2% alhydrogel adjuvant (Invitrogen, Brenntag Biosector, Denmark) was used for immunization and blood were collected at 1 week after the last immunization.

Scrub typhus patient sera were used for staining *O. tsutusgamushi* in this study. Human sera were prepared from scrub typhus patients, control patients with an acute febrile illness not diagnosed as scrub typhus, or healthy volunteers, following institutional review board approval and the receipt of informed consent from all subjects. Horseradish peroxidase (HRP)-conjugated anti-mouse or anti-human IgG secondary antibodies (Thermo Fisher Scientific, MA, United States) were used for immunostaining. The anti-*E. coli* antibody (Abcam, Cambridge, MA, United States) was used for visualizing the association of *E. coli* with cells. The Alexa Fluor 488‐ or Alexa Fluor 594-conjugated anti-mouse, -human antibodies and Alexa Fluor 488 Phalloidin and TO-PRO3 used in the immunofluorescence assays were purchased from Molecular Probes (Thermo Fisher Scientific, MA, United States).

### Protein Binding Assay

HeLa cells (2 × 10^5^ cells in a 24-well plate) were incubated with glutathione S-transferase (GST) or GST-ScaB proteins for 1 h, washed extensively with PBS, and fixed with 4% paraformaldehyde for 15 min. Cells were subsequently incubated with ToPro-3 (Thermo Fisher Scientific, MA, United States) for nuclear staining and observed under a confocal microscope or analyzed using a BD LSRFortessa (Becton Dickinson, Mountain View, CA, United States).

### Membrane Fractionation of *E. coli*

The outer membrane of recombinant *E. coli* was fractionated as previously described (Cardwell and Martinez, 2009). Briefly, 10 ml of induced *E. coli* that harboring an empty vector or a vector encoding *scaB* gene (nucleotide 118 to 1947) cultures was pelleted and resuspended in 1 ml of lysis buffer (PBS plus protease inhibitor cocktail). Cells were lysed by sonication for 3 s and incubated further for 10 s on ice. Unbroken cells were removed by centrifugation for 10 min at 4°C at 1,000 × *g*. The supernatant was transferred to a new tube, and inner membrane protein was extracted with Sarkosyl (final concentration, 0.5%) at room temperature for 5 min. Outer membrane fractions were pelleted by centrifugation at 13,000 × *g* for 30 min at 4°C, resuspended in 2 X sample buffer, resolved by sodium dodecyl sulfate-polyacrylamide gel electrophoresis (SDS-PAGE), and subsequently analyzed by immunoblotting with anti-ScaB mouse serum and goat anti-mouse IgG-HRP conjugate.

### Immunofluorescence Microscopy

Immunofluorescence microscopy was used to visualize *O. tsutsugamushi*. L929 cells infected with *O. tsutsugamushi* were washed with PBS and fixed with 4% paraformaldehyde and then permeabilized with 0.2% Triton X-100. Pooled scrub typhus human serum or anti-ScaB immune serum for 1 h, followed by incubation with Alexa Fluor 488-conjugated goat anti-mouse IgG and Alexa Fluor 594-conjugated goat anti-human IgG (Thermo Fisher Scientific). In some experiments, recombinant *E. coli* was stained with preimmune mouse serum, anti-ScaB serum, or anti-*E. coli* followed by incubation with Alexa Fluor 488-conjugated goat anti-mouse IgG (Thermo Fisher Scientific). Alexa Fluor 488 Phalloidin and TO-PRO3 were used for staining the cell actin and nucleus. Cells were examined under an Olympus FV1000 laser scanning confocal microscope (Olympus, Tokyo, Japan). Images of cell sections were analyzed and processed using the Olympus Fluoview software (Olympus).

### Cellular Adhesion and Invasion Assay

Bacterial adhesion and invasion assays were performed as previously described (Cardwell and Martinez, 2009; Riley et al., 2010). Briefly, *E. coli* strains harboring an empty vector or a vector encoding *scaB* gene were induced with IPTG and added to confluent monolayers of HeLa, A549, and ECV304 cells in serum-free media. Portions of the bacterium-containing media were plated to determine the number of colony-forming unit (CFU) added to each host cell monolayer. Contact between bacteria and the cultured cells was synchronized by centrifugation at 200 × *g*, and the preparations were incubated at 37°C for either 20 or 60 min for the adherence and invasion assays, respectively. For the invasion assays, infected cells were washed extensively with PBS and incubated for 2 h with complete medium supplemented with 100 μg/ml of gentamicin to kill any extracellular bacteria. For all *E. coli* assays, infected cells were washed extensively with PBS and the bacteria liberated by incubation with 0.1% Triton X-100 in sterile water. The lysate was then plated on LB agar to enumerate the cell-associated bacteria. The results were expressed as the percentages of bacteria recovered relative to the number of bacteria in the initial inoculum.

For the inhibition assays, cells were preincubated with 50 μg/ml of GST or GST-ScaB_24–372_ at 37°C for 1 h. Thirty minutes after bacterial inoculation, infected cells were washed three times with PBS, fixed with 4% paraformaldehyde, and permeabilized in a 0.2% Triton X-100 solution for use in immunofluorescence assays incorporating scrub typhus patient serum followed by Alexa Fluor 488-conjugated goat anti-human IgG to stain host cell-associated bacteria. One hundred cells were randomly selected by using an Olympus FV1000 laser scanning confocal microscope and analyzed using the Fluoview software.

### Statistical Analysis

The data were analyzed using the Graph Pad Prism 5.01 software. Statistical analysis was performed using the two-tailed Student’s *t*-test with 95% confidence interval. Data were expressed as means ± SDs.

## Results

### ScaB Is Expressed in *O. tsutsugamushi*

**Figure 1:**
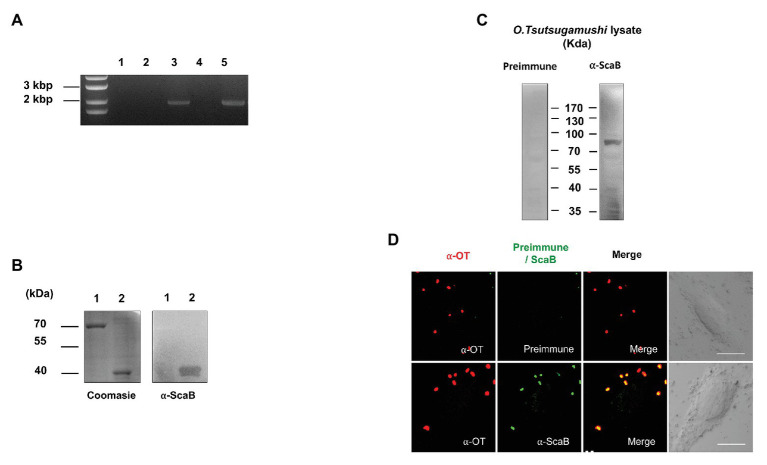
Expression of ScaB by *Orientia tsutsugamushi*. **(A)** Reverse transcriptase PCR (RT-PCR) of *scaB* mRNA from L929 cells infected with *O. tsutsugamushi*. Primer only without template (lane 1), total RNA (without reverse transcription) isolated from infected cells (lane 2), from genomic DNA (lane 3), from cDNA from uninfected cells (lane 4), and from cDNA from infected cells (lane 5). PCR product is 1950 bp in size. **(B)** Specificity of anti-ScaB antisera. BSA (lane 1) and recombinant ScaB protein (amino acids 23–1,372; lane 2) were stained with Coomassie blue (left panel) and immunoblotted with the anti-ScaB antisera. Recombinant ScaB protein is 36 kDa in size. **(C)** Western immunoblot analysis of *O. tsutsugamushi* whole-cell lysate probed with mouse preimmune sera (left panel) and anti-ScaB serum (right panel). Full-length ScaB protein is 77 kDa in size. **(D)** Immunofluorescence confocal microscopy using preimmune serum or anti-ScaB serum in the *O. tsutsugamushi*-infected L929 cells. The left-hand panels show bacteria stained with pooled serum from scrub typhus patients (∝-OT). DIC, differential interference contrast. Scale bars, 10 μm.

To determine whether *scaB* is actively transcribed in *O. tsutsugamushi* Boryong during interaction with host cells, we purified total RNA from bacteria-infected L929 cells, removed any contaminating bacterial or host genomic DNA, and performed RT-PCR to amplify the *scaB* transcripts. As shown in Figure 1A, the expression of specific *scaB* transcripts (1950 bp) was confirmed in *O. tsutsugamushi*. Next, to identify endogenous ScaB protein in *O. tsutsugamushi*, we generated a ScaB antiserum by immunization of mice with the soluble ScaB passenger domain (amino acids 36–373; Figure 1B). The *scaB* gene is predicted to encode a protein with a molecular mass of ~73 kDa. Western blot analysis of whole-cell lysates of *O. tsutsugamushi* using anti-ScaB serum showed a reactive band at ~80 kDa, which was not observed in non-immunized mouse serum (Figure 1C). To further confirm the cellular localization of ScaB in *O. tsutsugamushi*, bacteria were immunostained with pooled sera of scrub typhus patients together with anti-ScaB serum or preimmune mouse serum. As shown in Figure 1D, ScaB was observed on the surface of *O. tsutsugamushi* cells, but not when stained with the preimmune mouse serum control. Taken together, these results confirm that the *scaB* gene is actively transcribed and translated in *O. tsutsugamushi* and that the protein might be expressed on the bacterial outer membrane surface.

### ScaB Protein Mediates Adherence to and Invasion Into Host Cells

**Figure 2:**
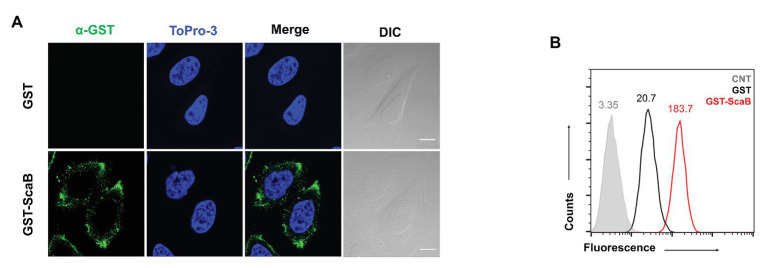
Recombinant ScaB protein-binding assay to HeLa cells. **(A)** Recombinant glutathione S-transferase (GST) or GST-ScaB_23–372_ protein was incubated with HeLa cells for 1 h. Cells were visualized by fluorescence microscopy after incubated with anti-GST followed by Alexa488-conjugated goat anti-mouse IgG antibody (green) and ToPro-3 (blue). Scale bars, 10 μm. **(B)** Flow cytometric analysis of the GST (black) and GST-ScaB (red) protein binding to HeLa cells. The gray histogram represents untreated cells

In previous studies (Ha et al., 2011, 2015), we reported that the passenger domain of ScaA and ScaC proteins from *O. tsutsugamushi* could mediate bacterial adherence to host cells by using the heterologous *E. coli* expression system. Here, we examined whether the *O. tsutsugamushi* ScaB protein is also involved in bacterial adherence or invasion. First, we performed a protein-binding assay using either purified GST or GST-ScaB soluble protein. Incubation of HeLa cells with GST-ScaB protein resulted in increased binding to the cells and punctate distribution in the cell membrane (Figure 2A). Binding efficiency was quantified by flow cytometry, and the mean fluorescence intensity (MFI) of the HeLa cells incubated with GST-ScaB was significantly increased (MFI = 183.7) compared to that of cells incubated with GST (MFI = 20.7) or that of untreated cells (MFI = 3.35; Figure 2B).

**Figure 3:**
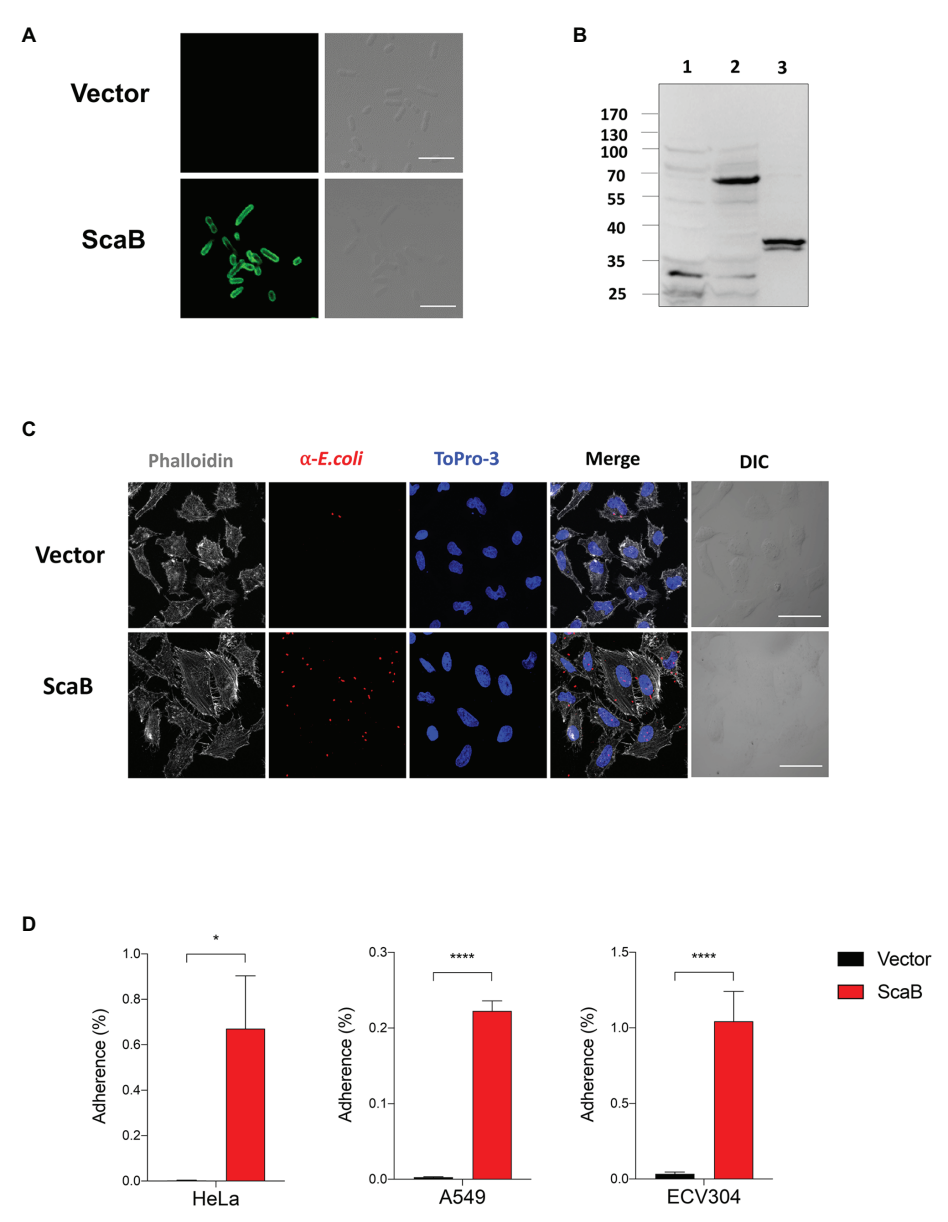
Expression of ScaB in *Escherichia coli* is sufficient to mediate adherence to mammalian cells. **(A)** Immunofluorescence microscopy using an anti-scaB antibody revealed the presence of ScaB on the surface of the recombinant *E. coli* (lower panel). Preimmune serum did not detect the recombinant protein (upper panel). Scale bars, 5 μm. **(B)** Expression of *O. tsutsugamushi* ScaB on the surface of *E. coli*. Immunoblot analysis of outer membrane fractions of induced *E. coli* harboring the empty vector (lane 1), ScaB (lane 2), and recombinant ScaB_23–372_ protein was performed using an anti-ScaB serum. **(C)** *E. coli* transformed with the pET28a vector or with ScaB domain was induced with isopropyl *β*-D-thiogalactoside (IPTG) and incubated with HeLa cells. Confluent monolayers of HeLa cells were infected for 30 min at 37°C, washed repeatedly with phosphate-buffered saline (PBS), and then stained with an anti-*E. coli* antibody (red), Phalloidin (gray), and ToPro-3 for nuclear staining (blue). Scale bars, 50 μm. **(D)** Colony-forming unit (CFU)-based quantification of adherent *E. coli* transformed with the vector (black bars) or ScaB (red bars) was performed for different host cells (HeLa, A549, and ECV304 cell lines). ^*^p < 0.05 and ^****^p < 0.0001. The data presented are representative of at least three independent assays for each cell line. Error bars represent the SD of each data set.

To further confirm the putative role of ScaB, the full length of the *scaB* open reading frame was cloned into an *E. coli* IPTG-inducible expression vector with a *pelB* signal sequence. Cellular localization and expression of ScaB were confirmed by confocal microscopy and immunoblotting with the anti-ScaB antibody. ScaB was observed on the surface of all recombinant *E. coli* cells, and a ~72 kDa product was confirmed in the isolated outer membrane fraction from ScaB-expressing *E. coli* (Figures 3A,B). Next, we assessed the ability of ScaB-expressing *E. coli* to adhere to cultured mammalian cells, including epithelial (HeLa and A549) and endothelial (ECV304) cells. Confluent monolayered cultured cells were incubated with IPTG-induced *E. coli* harboring either an empty vector or the ScaB-containing vector. Immunofluorescence analysis revealed an increase in the number of adherent ScaB-expressing *E. coli* bacteria (Figure 3C). This ScaB-mediated enhanced adhesion was verified by removing the adherent bacteria from the live host cells and counting them by CFU-based quantification assays. As a result, ScaB-expressing *E. coli* showed a significant increase in the adherence to both epithelial and endothelial cells (Figure 3D).

**Figure 4:**
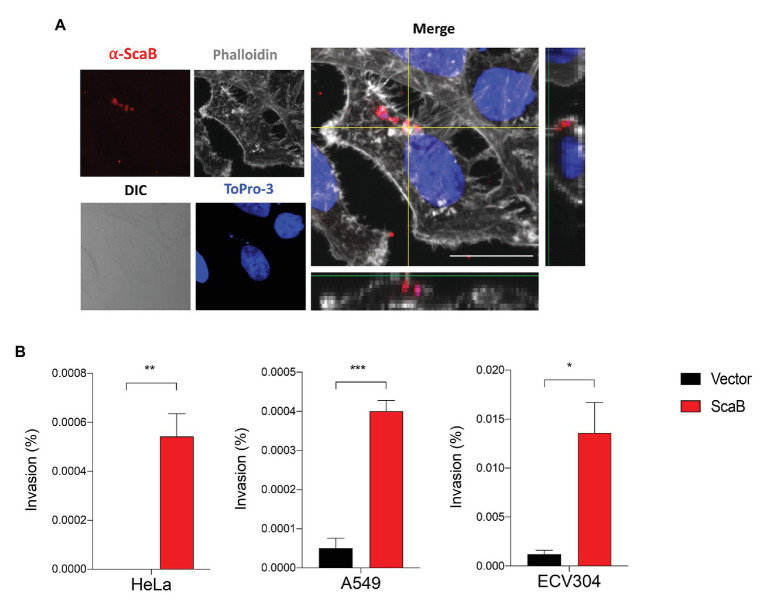
Expression of ScaB in *E. coli* is mediate invasion of mammalian cells. **(A)** Confluent cell monolayers of HeLa cells were infected for 1 h with *E. coli* transformed with the vector or ScaB and assessed for invasion by gentamicin protection assay. Cells were stained with an anti-scaB antisera (red), Phalloidin (gray), and ToPro-3 for nuclear (blue). The image is a projection of a 1 μm z-stack collected through x60 objective. Scale bars, 50 μm. **(B)** CFU-based quantification of bacterial invasion into mammalian host cells. Invasion is presented as the percentage of bacteria recovered after the gentamicin challenge out of the inoculums. The data presented are representative of at least two independent experiments for each individual cell line. Error bars represent the SD of each data set. ^*^p < 0.05, ^**^p < 0.01, and ^***^p < 0.001.

In a member of the nearest relative genus, *Rickettsia conorii*, OmpA (Sca0), OmpB (Sca5), and Sca2 have been shown to mediate not only adherence, but also invasion of host cells. To further confirm the ability of ScaB to mediate invasion of host cells, we utilized a gentamicin protection assay. Extracellular bacteria were killed by gentamicin, and internalized bacteria were quantified by CFU assays. As shown in Figure 4, in the absence of any *O. tsutsugamushi* surface antigens, ScaB is sufficient to mediate the invasion of either epithelial or endothelial cells. Taken together, these results demonstrate that unlike other *O. tsutsugamushi* Sca proteins (Ha et al., 2011, 2015), ScaB is involved in both adherence and invasion of host cells.

### The Soluble ScaB Polypeptide Inhibits Invasion by *O. Tsutsugamushi*

**Figure 5:**
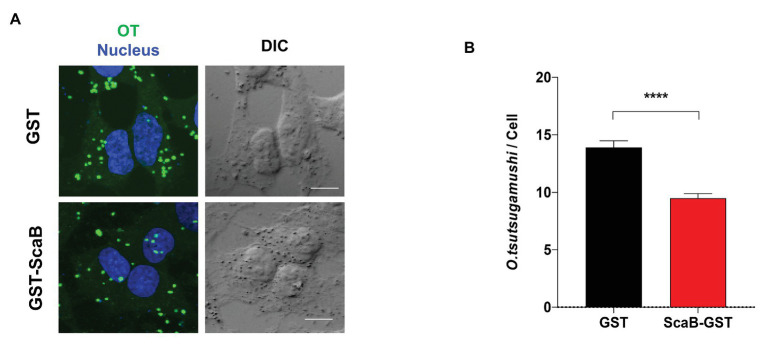
Inhibition of *O. tsutsugamushi* interactions with host cells by the ScaB polypeptide. **(A)** Preincubation of HeLa cells with GST-ScaB (lower panels) significantly inhibited *O. tsutsugamushi* interactions with host cells compared with preincubation with GST (upper panels). After incubation of the polypeptides with the host cells, *O. tsutsugamushi* was added, and incubation continued for a further 30 min. Cells were visualized by immunofluorescence microscopy. *Orientia tsutsugamushi* (green) and Nuclei (blue). **(B)** The numbers of bacteria associated with each of 100 randomly selected host cells. The bars indicate the means ± SDs of triplicate experiments. ^****^p < 0.0001.

In order to confirm the ScaB-mediated invasion of cultured mammalian cells, we further determined whether the soluble ScaB polypeptide could competitively inhibit *O. tsutsugamushi*-host cell interaction. HeLa cells were preincubated with 50 μg/ml of soluble GST or GST-ScaB polypeptide for 1 h, followed by incubation with *O. tsutsugamushi* for 30 min. Cells were extensively washed and the *O. tsutsugamushi*-host cell ratio was determined by confocal microscopy. As shown in Figure 5, preincubation with GST-ScaB induced a significant decrease in the total number of bacteria per cell [14 multiplicity of infection (MOI)/cell] compared to that of cells incubated with GST (9 MOI/cell). The number of bacteria per cell was reduced by approximately 35% when incubated with the GST-ScaB polypeptide compared to that of GST treated cells, suggesting that the soluble ScaB polypeptide competitively inhibits ScaB-mediated *O. tsutsugamushi* adherence to host cells by blocking its cognate mammalian ligand.

### Genetic Variation Among ScaB Genes From Different Strains of *O. tsutsugamushi*

**Figure 6:**
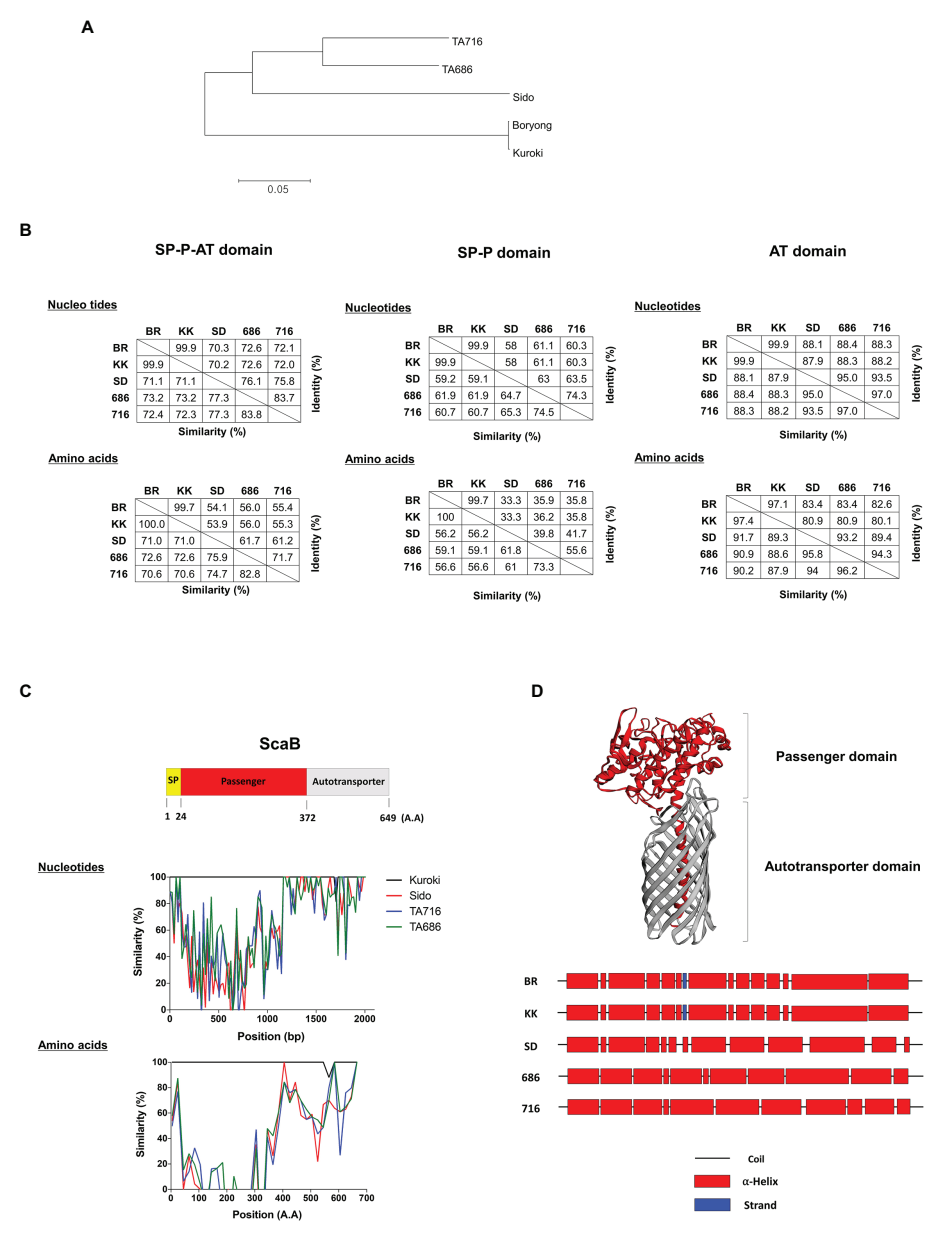
Comparison of *scaB* gene from indicated *O. tsutsugamushi* strains. **(A)** Amino acids sequence alignments for constructing phylogenetic trees were processed by Clustal W with the maximum likelihood method. **(B)** The similarity and identity of those nucleotides and amino acids were calculated through Matrix Global Alignment Tool [MatGAT; left: full-length of ScaB, middle: signal peptide(SP)-passenger(P) domain, and right: autotransporter (AT) domain]. **(C)** Similarity plot comparing ScaB sequence among the indicated *O. tsutsugamushi* strains and schematic representation above the graph indicates the relative sizes of translated ScaB amino acids sequence from Boryong strain and their sequence motiffs. **(D)** Secondary/tertiary structure of ScaB protein was predicted by HHPred, PSIPRED, and I-TASSER program. Predicted tertiary of ScaB protein (upper) and predicted secondary structure of passenger domain (Lower). BR, Boryong; KK, Kuroki; SD, Sido; 686, TA686; 716, TA716.

In a previous study using bioinformatics analysis, we reported duplicated *scaB* genes (GenBank accession no. CAM79930.1 and CAM81232.1) were only found in the Boryong strain; however, Koralur et al. (2018) also detected *scaB* in Sido and TA716 strains by tBLASTn. This prompted us to examine the presence or absence of *scaB* in various strains and to compare the genetic variation among the identified genes. Based on the complete *scaB* sequence from the Boryong strain, we performed conventional PCR with different *O. tsutsugamushi* genomic DNA samples using primer sets that cover the full-length or the passenger domain (Table 1). In addition, using tBLASTn with the Boryong ScaB protein sequence, we analyzed the presence of *scaB* genes from different partial *O. tsutsugamushi* genome sequences available in the NCBI database. As a result, we detected for the first time the full-length *scaB* gene in the Kuroki and TA686 strains by conventional PCR and tBLASTn. Comparison of the size of *scaB* genes showed highly conserved sizes with no significant differences among Boryong, Kuroki (1950 bp), Sido (1998 bp), TA686 (1971 bp), and TA716 (1995 bp) strains. To further investigate the sequence differences among the strains, we used various software tools to analyze the phylogenetic distance and sequence similarity and identity. As shown in Figure 6A, genetic variation among the strains was quite high. In the phylogenetic tree, two gene clusters were generated by maximum likelihood and the similarity and identity between the translated amino acid sequences of full-length ScaB were 70.6–100.0 and 53.9–99.7%, respectively. The sequence variation observed in ScaB is primarily attributed to the high level of sequence variation in the passenger domain (Figures 6B,C). The similarity and identity of the translated amino acid sequences of the passenger (P) domain were 56.2–100.0 and 33.3–99.7%, respectively, and of the autotransporter (AT) domain were 87.9–97.4 and 80.1–97.1%, respectively (Figure 6B). In addition, using a structural prediction program, we compared the secondary structure of each passenger domain. As shown in Figure 6D, it was characterized by 11–14 helices, but their locations were different. Along with similarities in the gene cluster, the Boryong and Kuroki strains also showed almost identical location of the ∝-helix; however, the Sido, TA686, and TA716 strains showed different patterns. Taken together, these results suggest that *scaB* is present in specific strains of *O. tsutsugamushi* and is highly variable among different strains, although it supports adhesion to or invasion into host cells.

## Discussion

Adherence to and invasion into host cells are essential steps of obligate intracellular bacteria and facilitate their persistence and survival in the host. These processes are mediated by multivalent ligand-receptor interactions (Pizarro-Cerda and Cossart, 2006). In this regard, *O. tsutsugamushi* must enter host cells to exploit their cellular machinery for intracellular replication. Although a precise understanding of host cell invasion mechanisms remains to be elucidated, several bacterial ligands for bacterial adhesion have been identified. Based on the genomic analysis of two *O. tsutsugamushi* strains, there are more than hundred *O. tsutsugamushi*-specific genes that are absent from the sequenced genomes of other species belonging to the family *Rickettsiaceae* (Cho et al., 2010a). Among the *O. tsutsugamushi*-specific genes, several outer membrane proteins, including those encoded by the *tsa56* and autotransporter genes, were shown to be expressed (Cho et al., 2007, 2010a; Nakayama et al., 2008). Previously, we reported that *O. tsutsugamushi* can bind to host fibronectin and utilize it for adherence or invasion *via* interactions with the outer membrane protein TSA56 (Lee et al., 2008). In addition, ScaA and ScaC were demonstrated to mediate adherence to mammalian host cells and could be used for diagnosis and applied as potential vaccine antigens. However, the biological role and immunogenicity of other Sca proteins have not been elucidated yet.

In this study, we investigated the role of ScaB during bacterial infection and analyzed the sequence conservation among various strains. Due to the challenges in the genetic manipulation of *O. tsutsugamushi*, we utilized the surrogate *E. coli* expression system that has been successfully used to characterize the rOmpB, Sca1, and Sca2 functions of *R. conorii* (Cardwell and Martinez, 2009; Chan et al., 2009; Riley et al., 2010). Using ScaB-expressing *E. coli*, we demonstrated that ScaB is sufficient to mediate adherence to and invasion into cultured epithelial and endothelial cells (Figures 3A,B). During the early stages of infection, *O. tsutsugamushi* exploits integrin-mediated signaling that induces local actin rearrangement at the site of infection (Cho et al., 2010b). It has also been shown that the Sca2 autotransporter protein of *Rickettsia conorii* mimics eukaryotic formins, generating long and unbranched actin filaments by combining nucleation and progressive barbed-end elongation activities (Cardwell and Martinez, 2012). As indicated by our immunofluorescence microscopy data (Figure 3C), ScaB-expressing *E. coli* did not induce actin rearrangement at the adhesion site, suggesting that ScaB may simply increase bacterial affinity to host cells. Nevertheless, preincubation of mammalian cells with recombinant ScaB polypeptide reduced *O. tsutsugamushi* invasion (Figure 5), suggesting that ScaB may function in the initial stage of *O. tsutsugamushi* adhesion and support the invasion process mediated by other bacterial virulence factors. The mammalian receptor involved in ScaB-mediated adhesion/invasion needs to be identified in future studies.

Here, we also detected the *scaB* gene in Kuroki and TA686 strains by searching the ScaB protein sequence and confirmed its presence by conventional PCR. The sequence analysis of full-length *scab* and the genetic variation among the five strains, Boryong, Kuroki, Sido, TA716, and TA686, indicated that the protein encoded by this gene possesses high levels of amino acid sequence variation in the passenger domains, showing variable similarity and identity of 70.6–100.0 and 53.9–99.7%, respectively (Figure 6). Considering the variation in the passenger domains potentially involved in the interaction with the environment, ScaB proteins in different strains may endow differential ability to adhere to and invade host cells. For example, UpaH, a virulence factor of uropathogenic *E. coli* (UPEC), is an autotransporter protein that mediates adherence to human extracellular matrix protein. Amino acid sequences of UpaH are highly variable among different UPEC strains, and their sequence variation is associated with differences in the abilities of different strains to mediate host adherence and biofilm formation (Allsopp et al., 2012). Therefore, functional differences in ScaB-mediated host cell adhesion and invasion among different strains could be an interesting subject for future studies.

Recently, a survey on *sca* prevalence was performed on 178 *O. tsutsugamushi* DNA samples collected from 12 endemic countries from diverse geographical areas; only *scaA*, *scaC*, *scaD*, and *scaE* were widely detected (93.8–100.0%). In contrast, *scaB* and *scaF* were detected less frequently (33.7–43.3%) and in samples from a limited number of countries. Interestingly, most DNA samples from Australia were positive for *scaB*. Bioinformatic analysis of species of *Rickettsia*, which forms the sister clade of *Orientia*, showed that at least 17 autotransporter genes, denoted as surface cell antigen (*sca*) genes, are present in the bacterial genome, and five of the *sca* genes, including *ompA* (*sca0*), *sca1*, *sca2*, *sca4*, and *ompB* (*sca* 5), have evolved under positive selection and are present in the genomes of most rickettsial species (Blanc et al., 2005). Among the autotransporter genes from *O. tsutsugamushi*, *scaA* and *scaE* have evolved under positive selection (Koralur et al., 2018). Except for the Boryong and Ikeda strains, the genome sequences of other *O. tsutsugamushi* strains in the databases were incomplete and comprised many contigs. Therefore, it is not possible to confirm whether the *scaB* gene is actually absent from their genomes or is only absent in the available incomplete assemblies. Since a limited number of *scaB* gene sequences are available, evolutionary pressure on the *scaB* gene can only be assessed after a sufficient number of sequences are available. Furthermore, the presence of *scaB* in diverse strains and its correlation with disease severity need to be investigated further.

Currently, the selection of the proper antigens is one of the critical barriers to generating cross-protective immunity against antigenically diverse strains of *O. tsutsugamushi* (Kelly et al., 2009). Thus far, TSA56 has been the best antigen to provide protective immunity in mouse infection models, but only against homologous strains. Previously, we demonstrated that immunization with combined TSA56 and ScaA autotransporter proteins significantly enhanced protective immunity against heterologous strains (Ha et al., 2015). The potential applicability of the ScaB antigen as a vaccine candidate can be also studied in animal infection models.

To our knowledge, similar to NadA from *Neisseria meningitidis* (Capecchi et al., 2005) and YadA (Heise and Dersch, 2006) from enteropathogenic *Yersinia* species, the ScaB protein of *O. tsutsugamushi* may mediate both adhesion and invasion of host cells and play important roles in bacterial pathogenesis. ScaB-expressing *E. coli* can adhere to and invade host cells in the absence of other *O. tsutsugamushi* genes. Moreover, the presence of a recombinant ScaB polypeptide in infection media specifically inhibits the association of *O. tsutsugamushi* with mammalian host cells. Functional characterization of the *scaB* gene in various strains and the use of potential vaccine targets with TSA56 should also be performed to facilitate the development of effective vaccines against scrub typhus.

## Data Availability Statement

The datasets presented in this study can be found in online repositories. The names of the repository/repositories and accession number(s) can be found in the article/supplementary material.

## Ethics Statement

The studies involving human participants were reviewed and approved by Institutional Review Boards of Seoul National University Hospital. The patients/participants provided their written informed consent to participate in this study. The animal study was reviewed and approved by Seoul National University Hospital Institutional.

## Author Contributions

YN and CK with the assistance of H-iK performed the majority of the experiments and analyzed the data. KJ generated bioinformatics data. YK, N-YH, and N-HC confirmed the data and wrote the manuscript. YN, CK, and YK contributed equally to this manuscript and should be considered equally contributing first authors. All authors contributed to the article and approved the submitted version.

### Conflict of Interest

The authors declare that the research was conducted in the absence of any commercial or financial relationships that could be construed as a potential conflict of interest.

## Footnotes

^1^http://www.r-project.org/

^2^http://toolkit.tuebingen.mpg.de/hhpred

^3^http://bioinf.cs.ucl.ac.uk

^4^http://zhanglab.ccmb.med.umich.edu/I-TASSER
